# Comparison of the precursor, amino acid oxidation, and end-product methods for the evaluation of protein turnover in senior dogs

**DOI:** 10.1371/journal.pone.0305073

**Published:** 2024-06-20

**Authors:** Letícia G. Pacheco, Camila Goloni, Ludmilla G. Di Santo, Lucas B. Scarpim, Débora A. Eugênio, Ariel de Castro, Vladimir E. Costa, Aulus C. Carciofi

**Affiliations:** 1 School of Agricultural and Veterinary Sciences, São Paulo State, University (UNESP), Jaboticabal, Brazil; 2 Institute Bioscience, Stable Isotope Center, São Paulo State University (UNESP), Botucatu, Brazil; Central South University of Forestry and Technology, CHINA

## Abstract

Stable isotope methods have been used to study protein metabolism in humans; however, there application in dogs has not been frequently explored. The present study compared the methods of precursor (13C-Leucine), end-products (15N-Glycine), and amino acid oxidation (13C-Phenylalanine) to determine the whole-body protein turnover rate in senior dogs. Six dogs (12.7 ± 2.6 years age, 13.6 ± 0.6 kg bodyweight) received a dry food diet for maintenance and were subjected to all the above-mentioned methods in succession. To establish 13C and 15N kinetics, according to different methodologies blood plasma, urine, and expired air were collected using a specifically designed mask. The volume of CO2 was determined using respirometry. The study included four methods viz. 13C-Leucine, 13C-Phenylalanine evaluated with expired air, 13C-Phenylalanine evaluated with urine, and 15N-Glycine, with six dogs (repetitions) per method. Data was subjected to variance analysis and means were compared using the Tukey test (P<0.05). In addition, the agreement between the methods was evaluated using Pearson correlation and Bland-Altman statistics. Protein synthesis (3.39 ± 0.33 g.kg-^0,75^. d^-1^), breakdown (3.26 ± 0.18 g.kg^-0.75^.d^-1^), and flux estimations were similar among the four methods of study (P>0.05). However, only 13C-Leucine and 13C-Phenylalanine (expired air) presented an elevated Pearson correlation and concordance. This suggested that caution should be applied while comparing the results with the other methodologies.

## Introduction

Stable isotopes are powerful tools that allow the study of protein metabolism without exposing animals to restriction and suffering, and without health or environmental contamination risks [1–3]. They have been routinely used in human nutrition [3–5]; however, although not new, the use of stable isotopes remains poorly explored in studies about dog nutrition [6].

Many nutritional, physiological, and pathological processes can interfere with protein turnover in adult animals, including diet composition [7], resistance exercise or hypertrophy [8,9], disease, and aging [10,11]. This process can induce changes in muscle mass, in turn, affecting health, physical activity, and disease resistance [10].

[12] published one of the first studies about protein metabolism in senior dogs and found an increase of approximately 50% in protein requirements of old beagles compared to adult beagles, although the nitrogen balance remained similar between compared age groups. These results were obtained using invasive markers of protein turnover such as muscle biopsy and changes in lean body mass. If metabolic tracers could be incorporated in such studies, valuable information about kinetics may be obtained. Therefore, turnover and metabolism of a particular nutrient of interest could be performed in a minimally invasive, precise, and “friendly” way, in turn, becoming a tool to better elucidate physiological issues such as the effect of ageing on protein metabolism [13].

Methods for precursor, end-product, and amino acid oxidation are well established in animal models to determine the rates of turnover, synthesis, and overall protein breakdown [14–16]. These methods can also be used to study body demand of amino acids [17–19]. The precursor method uses stable isotope ^13^C-Leucine. In addition to other essential amino acids that are metabolized in the liver, leucine is primarily catabolized in the muscle, well-known body protein reserve and participates in muscle protein synthesis, breakdown, and oxidative metabolism [20]. The final product method involves the use of ^15^N-Glycine, the first isotope tracer used to determine body protein turnover *in vivo* [21]. Some potential limitations to applying this method to dogs include the need for total urine collection. In addition, ^15^N in the tracer is incorporated differently based on various amino acids, and different turnover rates are observed when using other ^15^N-labelled amino acids [3,22,23].

Finally, an indirect method of amino acid oxidation, with ^13^C-Phenylalanine as a tracer, was developed with the aim of obtaining a minimally invasive model to study amino acid kinetics and its requirements, including studies in high-risk groups such as pregnant women, nursing mothers, the elderly, and newborns [2]. This approach involves the association of the precursor and final product methods, wherein urine is collected to quantify oxidation instead of plasma [2]. The isotope delivery can be either intravenous or oral, as the results were consistent in both delivery methods even though the route of administration is known to affect the tracer kinetics [24]. This method was originally developed to study amino acid requirements, but it may be used to obtain general values of protein flow, and determination of protein turnover. The rates of ^13^C-Phenylalanine incorporation into body reserves or oxidation in the postprandial period can be assessed using urine or expired air [25]; however, both approaches should be evaluated for advantages or disadvantages.

Considering that few studies are currently available that demonstrate usefulness in dogs of the methods of final products, indirect amino acid oxidation, and a radioactive isotope alternative for the precursor method [6,26,27], and that a direct comparison of results between them is not available, the present study compared the methods of precursor, end-product, and amino acid oxidation to determine the protein turnover rate in senior dogs that were fed a kibble diet for maintenance. In addition to comparing the results, the methods were standardized for their practical aspects related to feasibility and their practical application in studies with laboratory or privately owned dogs.

## Material and methods

The study was conducted at the Research Laboratory of Nutrition and Nutritional Diseases of Dogs and Cats “Prof. Dr. Flávio Prada”, Universidade Estadual Paulista (UNESP), Jaboticabal, Brazil. All procedures were approved by the Ethics Committee on the Use of Animals (CEUA) at the institution (protocol number 009537/18).

### Animals, housing and experimental design

**Fig 1:**
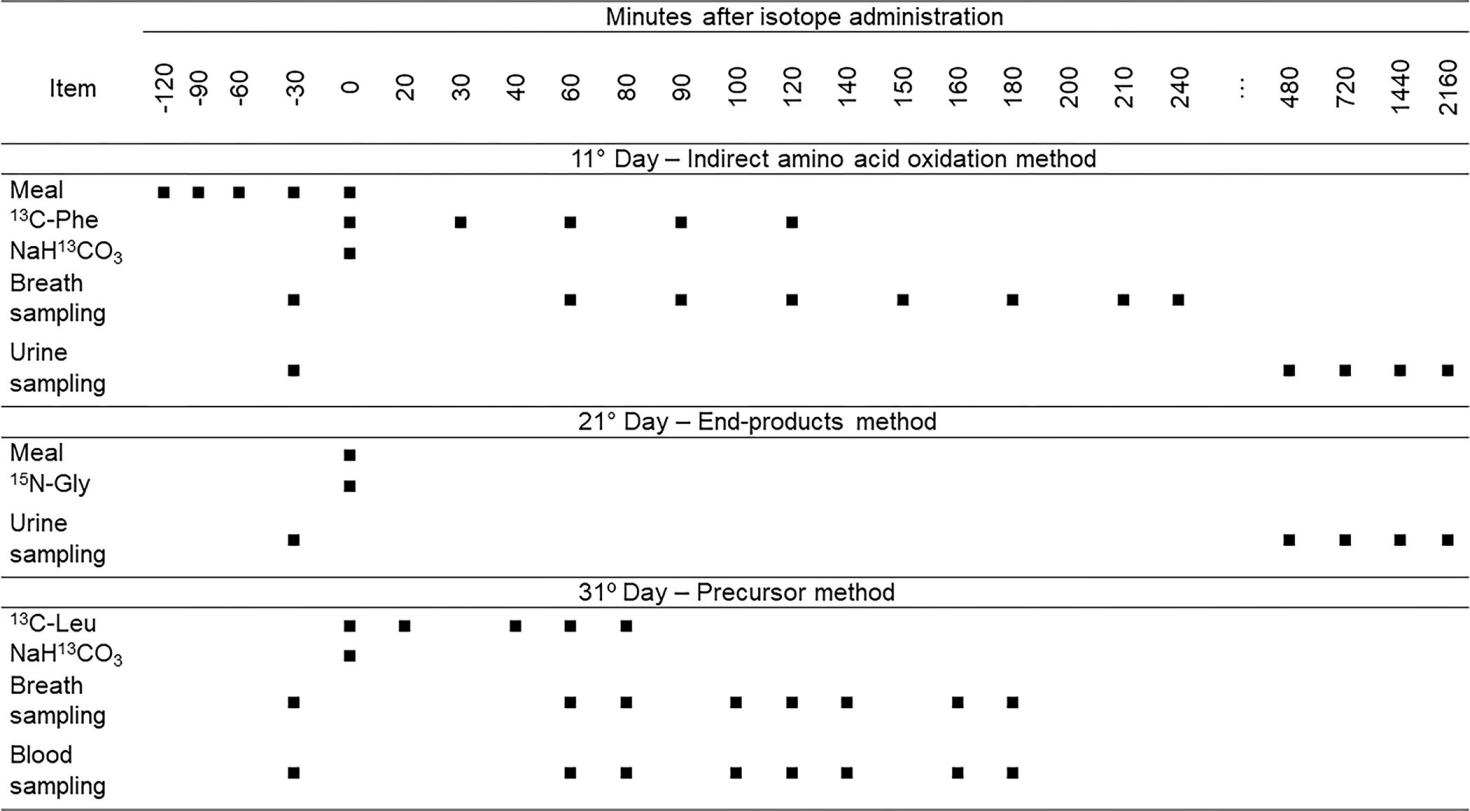
Enrichment and sampling protocol adopted in the indirect amino acid oxidation (^13^C-Phenylalanine), end-products (^15^N-Glycine) and precursor (^13^C-Leucine) methods.

The health of all dogs was previously verified by a veterinarian through physical examination, haemogram, and serum biochemical analyses, and all dogs were considered healthy. Six beagles (12.7 ± 2.6 years age and 13.6 ± 0.6 kg bodyweight) were fed with an extruded diet in controlled amounts to maintain a constant body weight (Natural Senior, Guabi Nutrição e Saúde Animal LTDA, Campinas, Brazil. Analysed chemical composition: 26% crude protein, 13% crude fat; 4.5% crude fiber; 1.92% leucine; 0.99% phenylalanine; 0.74% tyrosine; 1.83% glycine (DM basis); 16.1 kJ.g^-1^ of metabolisable energy). Dogs received the food for 35 days and first days i.e., days 1 to 10, were considered for adaptation to the diet. Food amount was adjusted to animals maintain a constant body weight, to this the energy requirement was stablished based on the intake records of each individual, and the amount provided established considering the food metabolizable energy content. Dogs were then weekly weighted at fasting in the morning, and the amount supplied adjusted if necessary. The indirect amino acid oxidation method, the end-products method, and the precursor method were conducted on day 11, day 21, and on day 31, respectively. On Day 35, the volume of CO_2_ produced by each dog was measured using a respirometer. The enrichment and sampling protocols used for the three methods are illustrated in Fig 1.

### ^13^C-Phenylalanine

For this test, dogs were housed individually in metabolic cages equipped with an apparatus to separate feces and urine for collection, measuring 0.9 m × 0.9 m × 0.9 m. All dogs were previously trained to stay in the cages. They underwent behavioral enrichment while in the cages, and one person stayed in the cage room most of the day. After 24 h fasting, the animals were fed their daily meal, but this total daily amount was divided into five equal portions, and each portion was offered at intervals of 30 minutes (Fig 1). So, the daily food amount was divided into five similar meals fed at each 30 min. Only dogs that consumed all the offered food in all the five meals were tested. Dogs that did not complete a meal were repeated in the following day.

Immediately after completing the fifth meal, dogs received a priming oral dose of 0.7 mg.kg^-1^of L-[1-^13^C]-Phenylalanine and 0.2 mg.kg^-1^ of NaH^13^CO_3_ (Cambridge Isotope Laboratories, Tewksbury, US). For administration, both isotopes were previously conditioned in gelatin capsules. After this prime dose, the dogs were orally supplied for four times, at every 30 minutes, gelatin capsules with 1.2 mg.kg^-1^ of L-[1-^13^C]-Phenylalanine.

Expired air samples were obtained both before the enrichment (basal state, collected before any isotope administration) and at specific intervals of time computed after the priming dose, namely: after 60, 90, 120, 150, 180, 210, and 240 minutes. During each sampling time, expired air was collected for a minimum of 2 minutes. The collection was conducted using a specially designed mask for the study, directly into a glass vial attached to the mask. The collected samples were then stored at room temperature for subsequent analysis.

**Fig 2:**
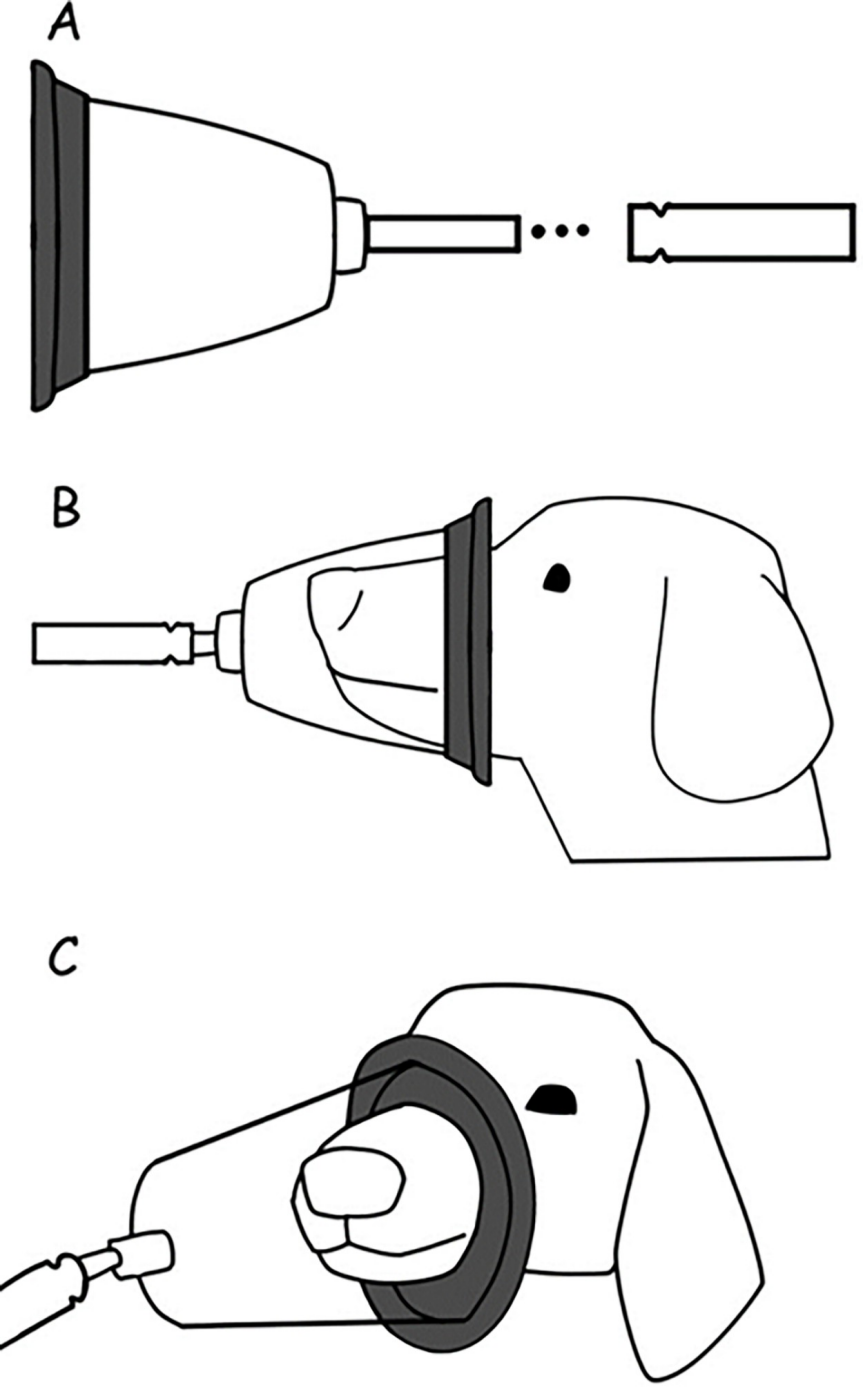
Illustration of the mask designed to collect breathed air samples in the study. A. Illustration of the rubber membrane, the mask, and the glass vial. B. Illustration of the mask positioned in the dogs’ nose, completely covering the mouth of the animal. C. Front view of the mask positioned during the air sampling procedure.

The masks were developed by the researchers and designed to cover the entire nose and mouth of the dogs, to prevent expired air from escaping. A rubber membrane at the end attached the mask to animals’ skin, to the air cannot escape or entry from the bottom of the mask (Fig 2). All dogs were previously trained to breathe inside the mask without apparent discomfort. For this purpose, dogs were encouraged to voluntarily place their nose and mouth on the mask using wet food and positive reinforcement. When they lost fear of wearing a mask, they were further adapted for the necessary time breathing for data collection without signs of discomfort.

Expired air collection through masks has been previously validated. To this, after trained in the use of the mask, dogs was enrichment with 1.2 mg.kg^-1^ of L-[1-^13^C]-Phenylalanine. Expired air samples of the dogs was collected before and after body enrichment. The masks and collection procedures were considered sufficiently adequate when clear enrichment, plateau, and reduction in ^13^CO_2_/^12^CO_2_ fraction enrichment were observed in the expired air samples. This procedure was repeated in several dogs until the protocol e equipment was considered adequate.

Urine samples were collected prior to enrichment, and after 8h, 12 h, 24 h, and 36 h of the fifth meal and priming dose, and frozen at -20°C until analysis. Urine samples were quantitatively collected in plastic containers placed under the collecting funnel of the metabolic cages, with 4 mL of 6N HCl as a preservative (Synth, LABSYNTH, Diadema, Brazil). Before analysis, urine samples were thawed and lyophilized (Thermo VLP200, Thermo Fisher, Massachusetts, USA).

### ^15^N-Glycine

The dogs were individually housed in metabolic cages as previously described. After 24 h fasting, the animals were fed their meal and allowed to eat for 20 min. Immediately after completing the meal, the animals were administered with a single dose of 20 mg.kg^-1^ [^15^N] glycine (Cambridge Isotope Laboratories, Tewksbury, US). The labelled compound was orally administered in gelatin capsules. Only dogs that ate all the offered food were tested; animals that did not complete the meal were evaluated the following day.

Urine samples were collected prior to enrichment (basal state, collected before any isotope administration), and after 8 h, 12 h, 24 h, and 36 h of the meal and frozen at -20°C until analysis (Fig 1). The samples were quantitatively collected in plastic containers placed under the collecting funnel of the metabolic cages, with 4 mL of 6N HCl as a preservative (Synth, LABSYNTH, Diadema, Brazil).

For analysis, urine samples were thawed at room temperature and the incorporation of ^15^N were assessed in both, urinary ammonia, and urea. Briefly, ammonia and urea in the urine samples were separated using cation exchange resin (AG50W-X8, Bio-Rad Laboratories, California, USA), as described previously [28,29]. In the method, ammonia was immobilized into the resin in a neutral solution. The urea remains in the supernatant and was then converted into ammonia using urease (Sigma Aldrich, Missouri, USA), that was subsequently also incorporated into the resin. Ammonia was extracted from the resin using a procedure previously described [28,29], lyophilized, weighed into tin capsules, and subjected to N isotopic analysis.

### ^13^C-Leucine

On the test day, the cephalic vein was catheterized (Descarpack, São Paulo, Brasil) and a priming dose of a combination of 2 mg.kg^-1^ of L-[1-^13^C] Leucine and 0.1 mg.kg^-1^ of [13C] NaH^13^CO_3_ (Cambridge Isotope Laboratories, Tewksbury, US) was infused in dogs after 24 h fasting. After five minutes of this priming dose, a bolus infusion of 0.7 mg.kg^-1^ of L-[1-^13^C] Leucine was given four times through the catheter at every 20 min for, for a total period of 80 min.

Samples of expired air and blood plasma were collected prior to enrichment (basal state, collected before any isotope administration) and at 60, 80, 100, 120, 140, 160, and 180 min after prime dose enrichment. At each sampling, expired air was collected, for at least 2 min, directly into a glass vial coupled to the same mask as previously described and stored at room temperature for later analysis. Blood samples were collected using the catheter and a syringe, deposited in heparinized tubes, centrifuged for plasma separation and the plasma was kept frozen at -20°C. Immediately before analysis, plasma samples were lyophilized (Thermo VLP200, ThermoFisher, Massachusetts, USA).

### Determination of CO_2_ volume in an indirect calorimetry system

A respirometry system (Animal Respirometry System; Sable System International, Las Vegas, USA) was used to determine the volume of CO_2_ (VCO_2_) for each dog. The dogs were housed in hermetically sealed chambers, with dimensions of 1 m x 1 m x 1 m, with internal temperature and moisture control (ALB 1000 CG Respirometry Chamber; Inbras Equipamentos para Saúde, Ribeirão Preto, Brazil). The chamber air temperature and relative humidity were adjusted to 26±1°C and 55% to 60%, respectively. The dogs were previously trained to stay and feed inside the chambers for several days. They were subjected to behavioral enrichment and toys while inside the chambers, and one person stayed in the room the entire time while the dogs were in the respirometry system.

On the day of the experimentation, the dogs were fasted for 24 h, placed in the chambers, and fed their standardized meal. Water was provided *ad libitum*. Each experiment lasted 8 h. Based on previous data from pilot studies, 2 h were allowed to equilibrate the gases inside the chambers. Ambient air flows were introduced into the chambers using a negative pressure system with an average flow rate of 10 L min^-1^, with control and adjustments provided by a mass-flow pump with a maximum flow capacity of 100 L min^-1^ (Sable Systems International, Las Vegas, Nevada, USA). The air flow volumes were established to maintain CO_2_ saturations below 1.0% (average CO_2_: 0.3±0.1% and average O_2_: 20.4±0.2%) inside the chambers. Channel air sampling was performed using independent pressure gauges (RM-8 gas-flow multiplexer; Sable Systems International, Las Vegas, Nevada, USA). The air samples were routed by a subsampler (SS-4 Gas Analyzer Subsampler, Sable Systems International, Las Vegas, Nevada, USA) to a humidity meter (RH-300 Relative Humidity & Dew Point Analyzer, Sable Systems International, Las Vegas, Nevada, USA). Oxygen concentrations were measured using a paramagnetic analyzer (PA-10, Sable System International, Las Vegas, Nevada, USA), and CO_2_ concentrations were measured using a carbon dioxide analyzer (CA-10, Sable System International, Las Vegas, USA). Before measuring the gas concentrations, moisture was removed from the air using anhydrous magnesium perchlorate (Exodus Científica, Sumaré, São Paulo, Brazil). The control and gauging of the gas meters were performed using a standard air mixture (CO_2_: 1.0031% and O_2_: 21.00%; White Martins, Vinhedo, São Paulo, Brazil). The data obtained using a mass flow meter and gas analyzer were collected using an ExpeData system (Sable System International, Las Vegas, Nevada, USA).

The VCO_2_ produced for each dog was determined using the following equation [30]:

$${VCO}_{2(mL∙{min}^{-1})}={FR}_{e}\frac{\left[\left(F_{e}{CO}_{2}-F_{i}{CO}_{2})+F_{i}{CO}_{2}(F_{i}O_{2}-F_{e}O_{2}\right)\right]}{\left(1+F_{i}{CO}_{2}\right)}$$ (1)

where FR_e_ is the excurrent flow rate, F_e_ is the fractional concentration of gas in the excurrent airstream, and F_i_ is the concentration of gas in the incurrent airstream.

### Indirect calorimetry system validation

Before and at the end of the evaluations, the chambers and respirometry system were validated for their gas recovery efficiency by burning ethanol (Sigma-Aldrich, Missouri, USA) for 430 min, and using the combustion values proposed [31]. The percentage of recovery was established considering the volume of burned ethanol calculated (measuring in a scale its initial and final weight), the recorded inflow and outflow of air and the volumes of O^2^ and CO_2_ detected by the device [32].

### Isotopic analysis

The isotopic analyses were performed at the Stable Isotope Centre, Universidade Estadual Paulista (UNESP), Botucatu, Brazil. All samples were analyzed using continuous-flow isotope ratio mass spectrometry (CF-IRMS). The CO_2_ samples were analyzed using an automated breath ^13^C analyzer isotope ratio mass spectrometer (ABCA2; SerCon, Cheshire, UK). Briefly, the system extracts the sample from the tube, the Helium flow carries the sample to the CF-IRMS, and the *R*(^13^C/^12^C) isotopic ratio is determined and expressed as an atomic fraction x(^13^C) (%), according to the general equation described by [33]:

$$\chi {(}^{i}E)=\frac{R{{(}^{i}E{/}^{i}E)}_{\mathrm{sample}}}{1+R{{(}^{i}E{/}^{i}E)}_{\mathrm{sample}}}$$ (2)

where *R*(^i^E/^i^E) isotopic ratio was determined and expressed as the atomic fraction x(^i^E) (%).

The results were normalized using a single-point anchorage: standard versus working gas [34] using NBS-22 standards. The ABCA-IRMS uncertainty was estimated at ±0.16‰, according to [35]. The difference between x(^13^C)_final_ and x(^13^C)_initial_ was used to calculate the excess atom fraction x^E^(^13^C) (S1 Table).

Urine and plasma samples were weighed in 5 x 8 mm tin capsules (PN 24006400, Thermo Scientific, Germany) and analyzed using an elemental analyzer (EA)-type CF-IRMS. An IRMS (Delta V, Thermo Scientific, Germany) coupled with an EA (Flash EA, Thermo Scientific, Germany) and a gas interface (ConFlo IV, Thermo Scientific, Germany) were used.

The EA-IRMS uncertainty was estimated at ±0.15‰ and ±0.20‰ for δ^13^C and δ^15^N, respectively. The results were normalized by two-point anchorage (Paul et al., 2007) using IAEA-N-1 and USGS90 for δ^15^N. To calculate the excess atom fraction x^E^(^15^N), the difference between x(^15^N)_final_ and x(^15^N)_initial_ was utilized.

### Whole body protein turnover

The flux of the labelled compounds and the rates of protein synthesis and breakdown were calculated according to the model proposed by [21].

Q=O+S=I+B (3)

where Q is tracer flux (g.kg^-0.75^. d^-1^), O is the measured amino acid oxidation evaluated by expired gas (not considered when expired ^13^C was not measured), S is the protein synthesis rate (g.kg^-0.75^. d^-1^), I is the dietary N intake (g.kg^-1^. d^-1^), and B is the protein breakdown rate (g.kg^-0.75^. d^-1^).

First, the rate of ^13^CO_2_ released by the oxidation of the tracer, represented by the excreted fraction of ^13^CO_2_ (F^13^CO_2_: g.kg^-1^. day^-1^) was calculated as follows:

$${F^{13}O_{2}}_{\left({g.kg}^{-0.75}∙d^{-1}\right)}=\left(\frac{{VCO}_{2}∙{ECO}_{2}}{BW}\right)∙\left(\frac{60\times 41.6}{100}\right)MW∙1∙10^{6}∙24$$ (4)

where *VCO*_*2*_ is the rate of CO_2_ production of the animal as determined in respirometry chambers (mL.min^-1^), *ECO*_*2*_ is the ^13^CO_2_ enrichment plateau in the expired gas, BW is the body weight of the animal (kg). The constants 41.6 μmol.ml^-1^ and 60 min.h^-1^ convert *VCO*_*2*_ to mol.h^-1^ and the factor 100 changes the plateau to a fraction. *MW* is the molar weight of the tracer, converted into g by the multiplication of μg to 10^6^. The factor, 24, transforms hour in the day as required in the equation (Adapted from [20]).

Subsequently, the oxidation (O) of ^13^C-Phenylalanine (using expired air) and ^13^C-Leucine (using expired air) was calculated using the following equation:

$$O_{\left({g.kg}^{-0.75}∙d^{-1}\right)}=F^{13}CO_{2}\left(\frac{1}{x^{E}{\left(13C\right)}_{sample}}-\frac{1}{x^{E}{\left(13C\right)}_{tracer}}\right)\times 100$$ (5)

where x^E^(^13^C)_tracer_ is the excess atom fraction of the tracer, x^E^(^13^C)_sample_ is the excess atom fraction of the sample, and represents enrichment in the steady state.

The oxidation (O) calculated with urine samples for ^15^N-Glycine and ^13^C-Phenylalanine tracers equals the flux (Q), provided the other parameters are calculated in the enrichment plateau [20]. The flux (Q) of ^13^C-Phenylalanine, ^13^C-Leucine (using plasma) and ^15^N-Glycine were calculated as:

$$Q_{tracer(g∙{kg}^{-0.75}∙d^{-1})}=D\left(\frac{x^{E}{\left(13C\right)}_{tracer}}{{a∙x}^{E}{\left(13C\right)}_{sample}}\right)MW∙24\times 10^{6}$$ (6)

$$Q_{Glycine(g∙{kg}^{-0.75}∙d^{-1})}=D\left(\frac{x^{E}{\left(15N\right)}_{Glycine}}{x^{E}{\left(15N\right)}_{Urine}}\right)MW∙24\times 10^{6}$$ (7)

where D is the tracer infusion rate (μmol.kg^-1^. h^-1^), *a* is a constant with value 1.33 for plasma and 1.0 for other samples, x^E^(^15^N)_Glycine_ is the excess atom fraction of ^15^N-Glycine, x^E^(^15^N)_urine_ is the excess atom fraction of a harmonic mean from urea and ammonia [36], and *MW* is the molar weight of the tracer. Unit μmol is transformed to g by multiplication by the MW and 10^6^ and hours in a day are accounted for by multiplication with 24 (Adapted from [20]).

Protein synthesis (S) for ^13^C-Leucine or ^13^C-Phenylalanine was calculated using the flux (Q) and oxidation (O). Considering the equation developed by [21], protein synthesis for ^15^N-Glycine may be considered equal to the flux as oxidation is not calculated. Protein breakdown (B) was calculated for glycine or phenylalanine (when the animals were fed), based on the difference between the flux (Q) of ^15^N-Glycine or ^13^C-Phenylalanine and the intake of the amino acids (I). In the case of ^13^C-Leucine, in fasting animals, the I equals the isotope dose [20].

### Statistical analysis

The results were evaluated using the equations, and protein flux, synthesis, and breakdown were calculated. After confirming the normality of the residue and variance homoscedasticity, the data were subjected to ANOVA in a completely randomized design, with four methods (treatments) and six experimental units (dogs) per treatment. Means were compared using Tukey’s test for verifying differences obtained with F test. The confidence intervals were calculated for assessing agreement between the results of the different methods as biases (mean difference ± 1.96 SD), according to [37], and the mean values were compared using the Pearson correlation, as a complementary statistic. Bland-Altman statistics and Pearson correlation were performed using R Software (PBC, Boston, MA, 2020), and the remaining analyses were performed using SAS software 9.1 using Proc MIXED (SAS Institute, Cary, NC, USA, 2003). P values of 0.05 were considered significant and P<0.1 was utilized as trend.

## Results

Validation of the indirect calorimetry system with ethanol produced a mean CO_2_ recovery rate of 96±6%, indicating accuracy. Therefore, the obtained VCO_2_ values can be considered reliable, with a mean value of 75.4±6.0 ml of CO_2_.min^-1^ for the experimental group. The mean O_2_ recovery rate was 82±5% and a mean energy expenditure (EE) of 406.6 ± 14.6 kJ.kg^-0.75^. d^-1^ was calculated for senior dogs using the observed VCO_2_ and VO_2_. The mean protein intake of the dogs for the study was 6.96 ± 0.26 g.kg^-0.75^. d^-1^, calculated using the food intake records and protein content of the diet.

**Fig 3:**
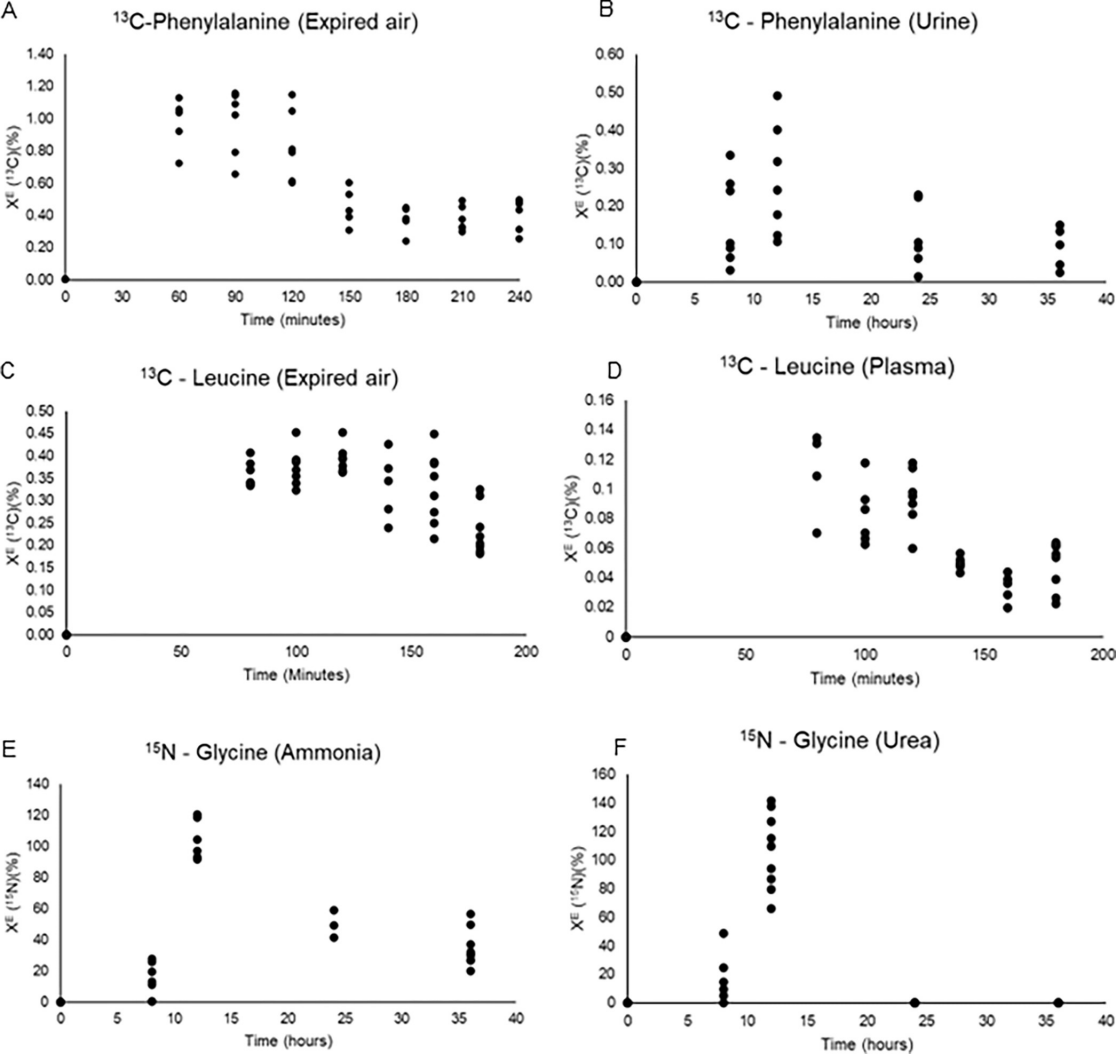
Fractional enrichment curve for each method of analysis: y = X^E^ (^13^C or ^15^N in %); x = sampling time in hours or minutes. A. ^13^C-Phenylalanine method; ^13^CO_2_/^12^CO_2_ evaluated along 200 min in expired air samples. B. ^13^C-Phenylalanine method; ^13^CO_2_/^12^CO_2_ evaluated along 36 hours in urine samples. C. ^13^C-Leucine method; ^13^CO_2_/^12^CO_2_ evaluated along 180 min in expired air samples. D. ^13^C-Leucine method; ^13^CO_2_/^12^CO_2_ evaluated along 180 min in plasma samples. E. ^15^N-Glycine method; ^15^N/^14^N evaluated along 36 hours in urinary ammonia samples. F. ^15^N-Glycine method; ^15^N/^14^N evaluated along 36 hours in urinary urea samples.

The isotope enrichment based on the different methods and biological samples is illustrated in Fig 3. As visible, it is possible to observe the enrichment curve for all the isotopes from which the tracer flux was calculated.

The F test of analysis of variance for all methods and biological samples resulted in similar estimates of protein synthesis, breakdown, and flux (P>0.05), showing comparable results between them, as presented in Table 1. The adopted procedure to collect samples of expired air, masks, and storage of the air samples in glass vials may be considered adequate, as the results calculated with plasma (^13^C-Leucine) and urine (^15^N-Glycine and ^13^C-Phenylalanine) were similar to those calculated with expired air samples (P>0.05). This indicates equally efficient rates of isotope recovery, or at least recoveries that are precise enough to result in similar estimations of the synthesis, breakdown, and flux of body proteins. The Bland–Altman statistics and Pearson correlation comparison between the rates of synthesis and breakdown of body proteins are presented in Table 2.

**Table 1:** Protein flux, synthesis, and breakdown rates in old beagle dogs obtained with different traces and biological samples.

Method	Flux	Synthesis	Breakdown
	(g of protein.kg^-0.75^.d^-1^)		
^13^C-Phenylalanine (expired air)	3.48	3.46	3.21
^13^C-Leucine (expired air and plasma)^1^	3.70	3.67	3.48
^15^N-Glycine (ammonia and urea)^2^	3.29	3.29	3.21
^13^C-Phenylalanine (urine)	2.91	2.91	2.90
SEM ^3^	0.35	0.16	0.15
P value	0.115	0.418	0.647

^1^ In the calculation procedure plasma and expired air atom percent in excess results are used to determine the parameters (Matthews et al., 1980) [20].

^2^ The atom percent in excess to calculate enrichment corresponds to the mean value obtained with urea and ammonia in urine [38].

^3^ SEM = standard error of the mean (n = 6 dogs per method).

**Table 2:** Pearson correlation and Bland & Altman statistics of the results obtained with the different methods and biological samples studied to estimate whole body protein synthesis and breakdown in dogs.

	Pearson Correlation	P value	Bias	Lower limit	Upper limit	Concordance Correlation Coefficient	Bias Correction Factor	R^2^
*Synthesis*			g.kg^-1^.d^-1^					
^13^C-Phe (expired air) x ^13^C-Phe (urine)	0.92	0.04	-0.07	-1.31	1.18	0.86	0.94	0.84
^13^C-Leu x ^13^C-Phe (expired air)	0.98	0.01	-0.10	-1.03	0.83	0.81	0.83	0.98
^13^C-Leu x ^13^C-Phe (urine)	0.61	0.14	0.13	-1.84	2.10	0.41	0.66	0.37
^13^C-Leu x ^15^N-Gly	0.82	0.09	0.74	0.07	1.42	0.33	0.40	0.68
^13^C-Phe (expired air) x ^15^N-Gly	0.51	0.33	0.65	-1.15	2.46	0.18	0.54	0.26
^13^C-Phe (urine) x ^15^N-Gly	0.74	0.23	-0.16	-1.05	0.73	0.68	0.91	0.55
*Breakdown*								
^13^C-Phen (expired air) x ^13^C-Phe (urine)	0.90	0.05	-0.31	-1.63	1.01	0.81	0.90	0.81
^13^C-Leu x ^13^C-Phe (expired air)	0.94	0.03	-0.04	-1.19	1.1	0.70	0.75	0.94
^13^C-Leu x ^13^C-Phe (urine)	0.45	0.22	-0.05	-2.22	2.12	0.26	0.57	0.20
^13^C-Leu x ^15^N-Gly	0.87	0.07	0.55	-0.03	1.14	0.47	0.54	0.75
^13^C-Phe (expired air) x ^15^N-Gly	0.53	0.32	0.52	-1.05	2.08	0.38	0.72	0.29
^13^C-Phe (urine) x ^15^N-Gly	0.85	0.18	-0.04	-0.91	0.83	0.77	0.91	0.72

Elevated Pearson correlation values were obtained for protein synthesis and breakdown, calculated using expired air or urine in the ^13^C-Phenylalanine method (P<0.05), with high concordance correlation coefficients, bias correction factors, and R^2^ values. This is expected as the same enrichment protocol and tracer were used. In addition, it confirms that both biological samples, the expired air and urine, were equally adequate for the procedure, in turn, validating the adopted collection protocol. The ^13^C-Leucine and ^13^C-Phenylalanine (expired air) methods also presented elevated Pearson correlation values for protein synthesis and breakdown (P<0.05), with elevated concordance correlation coefficients, bias correction factors, and R^2^ values, suggesting that both methods are interchangeable, and the selection of the most appropriate method depends on the experimental conditions and the moment (fasting or postprandial) at which protein metabolism will be studied. For the other methods, only a moderate Pearson correlation with a tendency of similar results (P<0.1) and moderate concordance correlation coefficient, bias correction factor, and R^2^ values were observed between ^13^C-Leucine and ^15^N-Glycine. Although a similar mean value was observed in the F-test, no Pearson correlation was observed (P>0.05) for the remaining methods.

## Discussion

The mean rates of protein synthesis, breakdown, and flux in the present study were consistent between the tested methods. The observed values were close to those for protein synthesis reported by [39] i.e., 3.08±0.13 g of protein.kg^-0.75^. d^-1^ in dogs fed a normal protein diet (15.1 g of crude protein per megajoule [MJ]). The diet used in the present study contained 16 g of crude protein per MJ, comparable to the diet used by [39], therefore justifying similar results. That authors evaluated protein metabolism through continuous infusion of ^13^C-Leucine in the blood as well as using plasma and expired air as sampling materials. Continuous intravenous infusion of ^13^C-Leucine for approximately two hours, with an enrichment plateau defined by arterial leucine, is the standard protocol for this tracer [20]. However, the comparison of [39] findings and those of the present study suggest that the protocol adopted for bolus enrichment with ^13^C-Leucine is adequate and comparable. Although bolus enrichment and frequent blood sampling necessitate the placement of a peripheral catheter, the procedure is easier and more “friendly” than a continuous infusion, which requires higher restriction and monitoring of dogs. In the authors’ experience, the proposed ^13^C-Leucine method is suitable for use in ambulatory conditions, with privately owned dogs after some dog training for blood sampling and collection of expired air.

The ^13^C-Leucine is considered the gold standard for determining protein turnover because of its true representation of free amino acid incorporation [40]. The calculated flux of labelled leucine was converted into whole-body turnover using the average leucine content of whole body protein (8 g/ 100 g protein, [20]). The difference between ^13^C-Leucine and the other two tracers is that the animals were fasting in the former, and although normal conditions of the animal’s daily life are not reflected, the measurements are adequate and reliable for most physiological conditions [3].

Whole-body protein turnover in adult and senior dogs was estimated by [6] using the ^15^N-Glycine tracer. Although the results varied considerably among diets and age groups, mean arbitrary values for dogs fed with 24% crude protein diet were 4.75 g and 4.35 g of protein degradation and synthesis per kg^-0.75^ per day, respectively. The authors found no significant difference between the age groups of dogs, suggesting that the measurements were influenced more by protein intake than by the physiological condition of the animals. The ^15^N-Glycine method was popular in the 1980s because of its non-invasiveness and easy application, as well as requirement of only a single dose of tracer and urine collection [22,41–43]. However, the application of this method presents some challenges outside the laboratory. Urine collection cannot be well-controlled and it may not be adequately sensitive for use in different physiological and pathological conditions [3]. While in humans, individuals may be requested to urinate, empty bladder before enrichment, and then urinate at the correct collection times, this is not possible in dogs, and the animals may have a residual amount of urine in the bladder before enrichment, resulting in dilution of the first point of the plateau (the first spontaneous voided urine of the animal). Additionally, dogs do not urinate at the appointed times but when they have an urge to, and the sampling time does not correspond with the required protocol. This issue has already been discussed in cats using the doubly labelled water method with urine as the sampling fluid [44] but, as in the present study, the final values were comparable, supporting the use of this tracer in dogs. To increase its precision, placing a urinary catheter to collect urine samples from dogs could be attempted; however, this is very invasive and beyond the scope of the present study.

One limitation of the use of ^15^N as a tracer for studies on protein metabolism is the assumption that all amino acids integrate into a single nitrogen compartment and simultaneously achieve the same enrichment [45]. However, this is not entirely true because amino acids may have specific and independent pathways of use and degradation [23]. Nitrogen breaks down into ammonia and urea, and the harmonic mean of ^15^N incorporation in the two compounds must be included for calculation [22,42], as adopted in the current study.

In the present study, the mean of synthesis, breakdown, and flux were similar between ^15^N-Glycine and the other two tracers, and the method showed a moderate correlation with ^13^C-Leucine, supporting the use of the proposed methodology to evaluate protein metabolism in dogs. Similar results were seen in a previous study in elderly humans which compared protein metabolism using ^15^N-Glycine and ^13^C-Leucine as metabolic tracers. The authors found slightly higher values of synthesis and degradation with ^13^C-Leucine in comparison to ^15^N-Glycine, but as in the present study, the differences were statistically insignificant [46].

The method utilizing ^13^C-Phenylalanine was originally developed to study amino acid requirements in a minimally invasive and precise approach [2,47,48]. Previous publications have studied amino acid requirements in various animals [49,50] and dogs [26,51]. The post-prandial oxidation or incorporation of ^13^C-Phenylalanine was determined in the fed state by measuring the fractional enrichment of ^13^CO_2_ (F^13^CO_2_) an animal enriched with the tracer. For the calculation, only F^13^CO_2_ is necessary, usually expressed as atoms percent in excess. The application of the method requires an equal body pool of phenylalanine and tyrosine in animals i.e., consuming equal amounts of these amino acids in excess of the requirement [52]. The diet used in the present study contained 0.99% phenylalanine and 0.74% tyrosine, well above the minimum requirements for these amino acids in dogs [53]. The authors decided to apply the tracer to estimate the protein flux and calculate the rates of whole protein synthesis and breakdown. To the best of our knowledge, this is the first publication that includes these data. Therefore, the basic parameter of F^13^CO_2_ was applied to the standard equations proposed for the other tracers, and whole-body protein metabolism was estimated. These values were found to be similar to those of the other tracers. The results of ^13^C-Phenylalanine calculated with expired air and ^13^C-Leucine showed high Pearson correlation, with low bias and a high concordance correlation coefficient and R^2^. Considering that most authors consider ^13^C-Leucine as a gold standard, results of the present study support high accuracy and precision of ^13^C-Phenylalanine (expired air) as a tracer to study protein metabolism, and not only to study amino acid requirements to animals, the original purpose for this method development.

The route of ^13^C-Phenylalanine delivery and the sample used to study ^13^C incorporation in animals were of concern, as they may interfere with the results [27,47]. [2] developed a 1-day protocol for protein metabolism studies comparing intravenous and oral infusion of ^13^C-Phenylalanine and analyzing the results in urine or expired air. Similar to the present study, the authors did not find any differences between the results of the tracer delivery route and the use of urine or expired air to assess ^13^C incorporation. However, although statistically similar, the results obtained for urine were slightly lower than those obtained for expired air in the present study. Additionally, no Pearson correlation, elevated bias, low concordance correlation coefficient, or R^2^ values were observed between ^13^C-Leucine and ^13^C-Phenylalanine using urine. Taking this into account and considering the previous discussion on the difficulty of collecting urine in a proper moment, caution is required when applying the ^13^C-Phenylalanine method with urine as a sampling material.

## Conclusions

The adopted procedures for precursor (^13^C-Leucine), end-products (^15^N-Glycine), and amino acid oxidation (^13^C-Phenylalanine) allowed similar results for protein synthesis, breakdown, and flux in senior dogs and can be used for research purposes in the future. The sampling procedures for expired air and urine were considered adequate, allowing the estimation of similar values for protein metabolism. However, only the results for ^13^C-Leucine and ^13^C-Phenylalanine (expired air) methods presented elevated Pearson correlation and concordance, suggesting caution when comparing the results of other methodologies. The selection of the most suitable method will depend on the experimental conditions, as ^13^C-Leucine evaluates animals in a fasting state while ^13^C-Phenylalanine and ^15^N-Glycine evaluate animals in fed state. In addition, the feasibility of total urine collection will depend on the structure of the research and dog population of the study.

## Supporting information

S1 Table Raw data to determine the whole-body protein turnover. Enrichment of samples in basal and plateau time, flux, synthesis and breakdown of animals.
(XLSX)

## Abbreviations

B: breakdow
F^13^CO_2_: fraction excretion of ^13^CO_2_
I: intake
O: oxidation
Q: flux
S: synthesis
VCO_2_: volume produced of CO_2_
δ^E^: relative difference between the isotopes in δ^E^ (‰)
χ^(E)^: excess atom fraction
